# Screening and Stratification Utility of GDF-15 and FGF-21 in Individuals Evaluated for Suspected Mitochondrial Disease: A Malaysian Cohort Study

**DOI:** 10.3390/metabo16060372

**Published:** 2026-05-29

**Authors:** Affandi Omar, Dyg Pertiwi Abg Kamaludin, Wan Ahmad Syazani Mohamed, Fatimah Diana Amin Nordin, Rosnani Mohamed, Badrul Hisyam Razali, Imilia Ismail, Ngu Lock Hock, Julaina Abdul Jalil

**Affiliations:** 1 Inborn Errors of Metabolism & Genetics Unit, Nutrition, Metabolic & Cardiovascular Research Centre, Institute for Medical Research, National Institutes of Health, Ministry of Health Malaysia, No 1, Persiaran Setia Murni U13/52, Bandar Setia Alam, Shah Alam 40170, Selangor, Malaysia; pertiwikamaludin1@gmail.com (D.P.A.K.); fatimahdiana@moh.gov.my (F.D.A.N.); rosnani.mohamed@moh.gov.my (R.M.); julaina.jalil@moh.gov.my (J.A.J.); 2 Nutrition Unit, Nutrition, Metabolic & Cardiovascular Research Centre, Institute for Medical Research, National Institutes of Health, Ministry of Health Malaysia, No 1, Persiaran Setia Murni U13/52, Bandar Setia Alam, Shah Alam 40170, Selangor, Malaysia; ahmad.syazani@moh.gov.my; 3 Institute of Medical Science Technology, Universiti Kuala Lumpur, Jalan TKS 1, Taman Kajang Sentral, Kajang 43000, Selangor, Malaysia; hisyamrazali@y7mail.com; 4 School of Biomedicine, Faculty of Health Sciences, Universiti Sultan Zainal Abidin (UniSZA), Gong Badak Campus, Kuala Nerus 21300, Terengganu, Malaysia; imilia@unisza.edu.my; 5 Genetics Department, Hospital Kuala Lumpur, Jalan Pahang, Kuala Lumpur 50586, Federal Territory of Kuala Lumpur, Malaysia; ngu.lockhock@moh.gov.my

**Keywords:** biomarker screening, FGF-21, GDF-15, inborn errors of metabolism, mitochondrial disease

## Abstract

**Background/Objectives**: Early detection of mitochondrial disorders remains challenging due to phenotypic heterogeneity and limited access to definitive molecular diagnostics. Circulating biomarkers such as growth differentiation factor-15 (GDF-15) and fibroblast growth factor-21 (FGF-21) have emerged as potential adjunct indicators. This study evaluated the screening and stratification utility of GDF-15 and FGF-21 in individuals assessed for suspected mitochondrial disease. **Methods**: Archived biological specimens collected between 2016 and 2017 were analysed from 221 individuals stratified into clinically high-risk, screen-positive non-high-risk, post-mortem unexplained death and healthy controls groups. Plasma and fibroblast lysate concentrations of GDF-15 and FGF-21 were quantified using enzyme-linked immunosorbent assays. Biomarker performance was assessed using receiver operating characteristic (ROC) analysis, comparative group analysis and correlation testing across clinically defined referral groups. **Results**: Both biomarkers were significantly elevated in clinically high-risk and screen-positive individuals compared with controls. GDF-15 demonstrated better discriminatory performance than FGF-21, with an area under the curve (AUC) of 0.7187 ± 0.0556 versus 0.6301 ± 0.0603. At a threshold of 300 pg/mL, GDF-15 demonstrated high sensitivity with moderate specificity for differentiation between clinically defined high-risk individuals and controls. Correlation analysis showed weak associations between GDF-15 and lactate and ammonia, while FGF-21 correlated modestly with glucose and alkaline phosphatase. A moderate positive correlation was observed between GDF-15 and FGF-21 across the overall cohort. **Conclusions**: GDF-15 and, to a lesser extent, FGF-21 may support early screening and stratification of individuals evaluated for suspected mitochondrial disease and assist in prioritising cases for further diagnostic evaluation.

## 1. Introduction

Inborn errors of metabolism (IEM) encompass a diverse group of genetic disorders characterised by disruptions in metabolic pathways that impair energy production, substrate turnover or cellular homeostasis. Among these, mitochondrial diseases represent one of the most diagnostically complex subgroups due to dual genomic control, variable inheritance patterns and multisystem clinical manifestations. Patients frequently present with heterogeneous neurological, muscular, cardiac or metabolic phenotypes, complicating early clinical recognition and delaying definitive diagnosis [1].

Current diagnostic workflows rely on integrated biochemical, histopathological and molecular investigations, including respiratory chain enzyme assays and genomic sequencing. However, access to advanced molecular diagnostics remains limited in many healthcare settings and definitive evaluation often requires invasive tissue sampling, particularly muscle biopsy, which poses logistical and ethical challenges. Consequently, there is sustained interest in minimally invasive biomarkers capable of supporting early suspicion, triage and prioritisation for confirmatory testing.

Circulating stress-responsive biomarkers have emerged as promising adjuncts in this context. Growth differentiation factor-15 (GDF-15), a member of the transforming growth factor-β superfamily, is induced in response to mitochondrial dysfunction, oxidative stress and integrated cellular stress signalling pathways [2]. Elevated circulating concentrations of GDF-15 have been consistently reported across primary mitochondrial disorders and are associated with disease severity and systemic metabolic stress responses [2].

On the other hand, fibroblast growth factor-21 (FGF-21), a metabolic regulator involved in lipid oxidation and mitochondrial energy homeostasis, is similarly upregulated in mitochondrial respiratory chain defects, particularly those affecting skeletal muscle. Recent comparative studies confirm that both biomarkers demonstrate diagnostic utility, although their performance varies by phenotype and disease context [3].

Large biomarker comparison analyses and contemporary reviews indicate that GDF-15 and FGF-21 currently represent the most informative circulating indicators of mitochondrial dysfunction, with GDF-15 often demonstrating slightly superior overall diagnostic performance [4,5]. Nonetheless, elevations may also occur in secondary mitochondrial dysfunction and systemic metabolic stress states, highlighting the need for contextual clinical interpretation [6].

Despite growing clinical adoption, most biomarker studies have focused on genetically confirmed mitochondrial cohorts. Data remain limited regarding performance across heterogeneous environments. Furthermore, the integration of circulating and cellular biomarker matrices in heterogeneous clinical cohorts remains insufficiently explored.

Advances in expanded newborn screening and metabolomics technologies have improved early detection frameworks for inborn errors of metabolism. Tandem mass spectrometry (MS/MS) and next-generation sequencing platforms now enable simultaneous interrogation of multiple metabolic pathways within population screening programmes [7,8]. However, mitochondrial disorders remain difficult to detect through conventional newborn screening because many patients do not present with consistent metabolite signatures during early disease stages. Recent studies have therefore explored metabolomic profiling and multi-omics integration as strategies to improve detection sensitivity for mitochondrial and energy metabolism disorders that may not be captured through conventional metabolite panels [9]. Despite these technological advances, mitochondrial diseases remain underrepresented in routine newborn screening algorithms due to phenotypic variability, delayed biochemical expression and limited disease-specific markers [10]. Consequently, there is ongoing interest in adjunct circulating biomarkers that may complement metabolomics-based detection and enhance early clinical suspicion.

Diagnostic access disparities further complicate early identification of mitochondrial disease across low- and middle-income regions, especially in Asia [11,12]. Limited availability of respiratory chain enzyme testing, genomic sequencing and specialised histopathology restricts timely confirmation of suspected cases [13]. In many settings, diagnostic workflows still rely on clinical phenotyping and basic biochemical assays, which may delay diagnosis and contribute to under-recognition of mitochondrial disease [14,15]. Thus, cost-effective, minimally invasive biomarkers capable of supporting referral triage and prioritisation for advanced testing therefore represent a practical interim strategy within resource-constrained healthcare systems. Evaluating biomarker performance within heterogeneous referral cohorts is particularly relevant to these real-world diagnostic environments.

This study therefore aimed to evaluate the screening and stratification utility of GDF-15 and FGF-21 in individuals assessed for suspected mitochondrial disease. Using archived plasma and fibroblast lysate specimens, we examined biomarker discriminatory performance using receiver operating characteristic (ROC) analysis and evaluated their relationships with selected biochemical parameters across clinically defined risk groups, including patients with confirmed oxidative phosphorylation deficiency.

## 2. Materials and Methods

### 2.1. Study Design and Patient Cohort

This retrospective study analysed archived serum, plasma and fibroblast specimens collected between 2016 and 2017 from individuals referred for evaluation of suspected mitochondrial disease at the Institute for Medical Research, Ministry of Health Malaysia. Referrals originated from tertiary hospitals throughout Malaysia and included patients presenting with clinical, biochemical or neuroimaging findings suggestive of impaired mitochondrial energy metabolism.

The operational definition of suspected mitochondrial disease in this study referred to individuals presenting with one or more features commonly associated with mitochondrial dysfunction. These included unexplained neurological manifestations, developmental regression, hypotonia, epilepsy, stroke-like episodes, encephalopathy, basal ganglia abnormalities, cardiomyopathy, lactic acidosis, recurrent metabolic decompensation or abnormalities detected through metabolic screening investigations.

Initial diagnostic evaluation was performed according to routine clinical practice and included combinations of plasma lactate, ammonia, amino acid analysis, urine organic acid analysis, acylcarnitine profiling, neuroimaging assessment and histopathological investigations when clinically indicated. Respiratory chain enzyme analysis, fibroblast studies and molecular investigations were performed in selected patients depending on sample availability and clinical suspicion.

A total of 221 individuals were included based on the availability of sufficient clinical and laboratory information for retrospective assessment. The study cohort was intentionally designed to reflect a heterogeneous real-world referral population rather than a genetically confirmed mitochondrial disease cohort alone.

**Figure 1:**
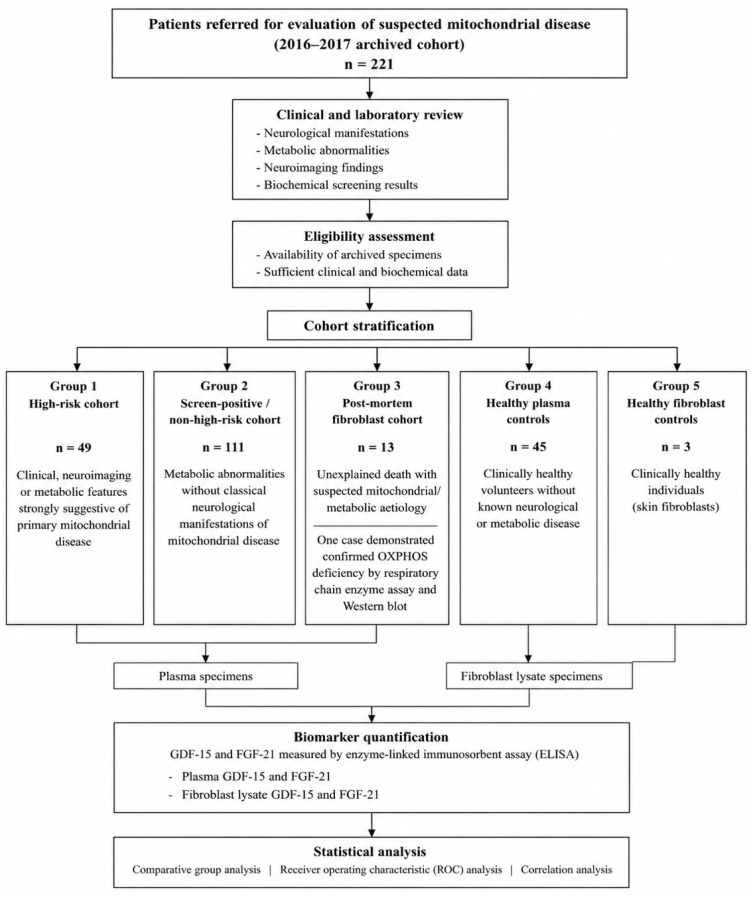
Study workflow and cohort stratification for evaluation of GDF-15 and FGF-21 in individuals assessed for suspected mitochondrial disease. Subjects were classified according to clinical presentation, biochemical findings and specimen availability prior to biomarker quantification and statistical analysis. One post-mortem fibroblast case demonstrated confirmed oxidative phosphorylation (OXPHOS) deficiency based on respiratory chain enzyme assay and Western blot analysis. Arrows indicate workflow progression and cohort classification steps.

Group classification was based on the degree of clinical suspicion for mitochondrial dysfunction and the presence of supportive biochemical or neuroimaging abnormalities identified during routine diagnostic evaluation. The clinically high-risk group included individuals with multiple features suggestive of mitochondrial disease, whereas the screen-positive/non-high-risk group comprised individuals with abnormal metabolic screening findings but without sufficient clinical features to meet high-risk criteria. Subjects were stratified into four principal clinical groups according to the predefined inclusion and exclusion criteria, as illustrated in Figure 1.

i. Group 1 (High-risk cohort) consisted of patients presenting with one or more clinical, neuroimaging or metabolic features suggestive of mitochondrial dysfunction (*n* = 49).
ii. Group 2 (Screen-positive/non-high-risk cohort) included individuals without classical neurological manifestations of mitochondrial disease but demonstrating biochemical abnormalities suggestive of impaired energy metabolism, including secondary lactic acidosis or abnormalities in amino acid, fatty acid oxidation or organic acid profiles (*n* = 111).
iii. Group 3 comprised post-mortem cases with unexplained cause of death in which an underlying mitochondrial or metabolic disorder was clinically suspected (*n* = 13).
iv. Group 4 consisted of clinically healthy volunteers without known metabolic or neurological disease who served as plasma healthy controls (*n* = 45).

In addition, skin fibroblast samples from three healthy individuals were included as Group 5 and served as cellular reference controls for fibroblast-based biomarker analyses. One post-mortem fibroblast case within Group 3 demonstrated confirmed oxidative phosphorylation (OXPHOS) deficiency based on respiratory chain enzyme assay and Western blot analysis performed on cultured skin fibroblast. Written informed consent was obtained from all participants and/or their parents/guardians prior to inclusion in the study.

Most individuals included in this study did not have molecular confirmation of mitochondrial disease. Therefore, the study cohort reflects a clinically heterogeneous referral population evaluated for suspected mitochondrial dysfunction rather than a genetically confirmed mitochondrial disease cohort. Consequently, biomarker performance analyses were interpreted as discrimination between clinically defined risk groups and controls rather than definitive diagnostic accuracy against a molecular gold standard.

### 2.2. Biological Sample Collection and Processing

Archived serum and plasma specimens from Groups 1, 2 and 4 were collected under fasting conditions according to sample availability within the retrospective cohort. Blood specimens were separated by centrifugation and stored at −80 °C until biomarker analysis. Serum and plasma samples were analysed using the same ELISA platform and interpreted collectively as circulating biomarker specimens.

Skin biopsies were obtained from Group 3 (post-mortem cases) and Group 5 (healthy fibroblast controls) to establish fibroblast cultures for cellular biomarker analysis. Tissue explants were established in a class II containment cabinet using standard fibroblast propagation techniques. Following expansion, fibroblast cells were harvested into cell pellets, lysed and stored at −80 °C in liquid nitrogen prior to downstream cellular analyses. Both plasma and fibroblast lysate specimens were analysed for GDF-15 and FGF-21 concentrations. Plasma samples were used for the primary receiver operating characteristic (ROC) and correlation analyses. Fibroblast lysate measurements were included to explore intracellular biomarker expression in post-mortem and cellular control samples, complementing the circulating biomarker analysis.

### 2.3. Quantification of GDF-15 and FGF-21

Biomarker concentrations were quantified using commercially available quantitative sandwich enzyme-linked immunosorbent assay kits (Quantikine^®^ ELISA DGD150 and Quantikine^®^ ELISA DF2100, R&D Systems, Minneapolis, MN, USA) according to the manufacturer’s protocols. Plasma and fibroblast lysate concentrations of GDF-15 and FGF-21 were measured using the Quantikine^®^ ELISA platform. Optical density was measured at 450 nm with wavelength correction at 540 nm or 570 nm using a calibrated Tecan Infinite 200 Pro microplate reader (Tecan Group Ltd., Mannedorf, Switzerland). Concentrations were derived through standard calibration curves generated using a four-parameter logistic (4-PL) model (GraphPad Software, Boston, MA, USA). All samples were analysed in triplicate. All assays were performed for research purposes and were not intended for clinical diagnostic use. The operator who performed the assay was blinded to the sample group allocation until completion of analysis.

### 2.4. Clinical Variable Definition

Clinical phenotypic variables were obtained from referral records and medical documentation. The variables were extracted for descriptive analysis and included epilepsy, septicaemia, organomegaly, muscle weakness, lethargy, hypoglycaemia, hypotonia, developmental delay, cardiomyopathy, intellectual disability and failure to thrive. All variables were coded as binary indicators (present = 1, absent = 2).

### 2.5. Statistical Analysis

SPSS Version 25 (IBM Corp., Armonk, NY, USA) was employed for statistical analyses. Continuous variables were expressed as mean ± standard deviation (SD) or median with interquartile range (IQR) depending on data distribution. Data distribution was assessed using the Shapiro–Wilk test. Biomarker concentrations demonstrated non-normal right-skewed distributions with wide interquartile ranges across most study groups. Therefore, biomarker levels were summarised using median and interquartile range (IQR) and non-parametric statistical methods were used for biomarker comparisons and correlation analyses where appropriate. For fibroblast control samples (Group 5), values were reported descriptively as mean ± standard deviation (SD) due to the small sample size and their use as exploratory cellular reference values. Comparisons between clinical groups were conducted using Student’s *t*-test or Mann–Whitney U test as appropriate.

Diagnostic performance was evaluated using ROC curve analysis and the area under the curve (AUC) (GraphPad Software, Boston, MA, USA). An AUC value of 1.0 indicates perfect discrimination, whereas an AUC of 0.5 indicates no diagnostic discrimination equivalent to random chance. The optimal biomarker cut-off thresholds were determined using Youden index optimisation. The reported cut-off thresholds for GDF-15 and FGF-21 were selected based on receiver operating characteristic (ROC) curve analysis using Youden index optimisation to achieve the best balance between sensitivity and specificity within the study cohort. Correlation between GDF-15 and FGF-21 concentrations and selected biochemical parameters was assessed using Spearman correlation coefficients depending on data normality. Statistical significance was defined as *p* < 0.05.

## 3. Results

### 3.1. Cohort Characteristics

A total of 221 individuals were included following clinical evaluation for suspected mitochondrial disease (Table 1). The high-risk group predominantly consisted of paediatric patients presenting with complex neurological features. Common clinical manifestations included stroke-like episodes, basal ganglia involvement, encephalopathy, hypotonia, epilepsy, myopathy and failure to thrive. These phenotypes constituted the primary basis for referral for mitochondrial disease evaluation.

### 3.2. Circulating Biomarker Concentrations

Baseline circulating biomarker concentrations in healthy controls demonstrated low circulating levels of both analytes, with median GDF-15 and FGF-21 concentrations of approximately 235 pg/mL and 39 pg/mL, respectively. Reference interval assessment in the healthy cohort showed GDF-15 values ranging from 97 to 713 pg/mL and FGF-21 ranged from 5 to 119 pg/mL, both demonstrating right-skewed distributions.

On the other hand, marked elevations were observed in clinically high-risk patients. The median of GDF-15 concentration reached approximately 4667 pg/mL, while the median of FGF-21 level was 203 pg/mL. These values were higher than those observed in health controls and may reflect increased systemic metabolic stress in clinically high-risk individuals. In the screen-positive but non-high-risk cohort, biomarker elevations were present but less pronounced. Median GDF-15 and FGF-21 concentrations were found to be approximately 319 pg/mL and 74 pg/mL, respectively, corresponding to approximately 2-fold increase above control levels. Fibroblast-based analysis was limited by the very small number of healthy control cell lines, which restricts statistical interpretation and increases susceptibility to variability. These findings should therefore be considered exploratory.

### 3.3. Comparative Group Analysis

Comparative statistical analysis demonstrated significant elevation of both GDF-15 and FGF-21 in high-risk patients relative to healthy controls (*p* < 0.05). Similarly, biomarker concentrations in screen-positive individuals were significantly higher than those of controls (*p* < 0.05). The magnitude of separation between clinical strata was greater for GDF-15 than for FGF-21, indicating stronger discriminatory behaviour across differing levels of disease suspicion. No significant differences were observed in biomarker concentrations by gender. In the healthy cohort, age showed a modest inverse association with GDF-15, although the effect size was small and of uncertain clinical significance.

### 3.4. Receiver Operating Characteristic Analysis

ROC analysis demonstrated better discriminatory performance for GDF-15 compared with FGF-21. The area under the ROC curve (AUC) for GDF-15 was 0.7187 ± 0.0556, indicating moderate discrimination between individuals with suspected mitochondrial disease and controls, whereas FGF-21 showed a lower AUC of 0.6301 ± 0.0603 (Table 2). This indicates moderate rather than definitive discriminatory performance. At a threshold of 300 pg/mL, GDF-15 demonstrated high sensitivity with moderate specificity for differentiation between clinically defined high-risk individuals and controls (*p* < 0.05).

Combined interpretation of GDF-15 and FGF-21 did not improve discriminatory performance compared with GDF-15 alone. The reduced performance observed in the combined analysis may reflect unequal contribution of the two biomarkers within this heterogeneous referral cohort. Therefore, interpretation focused primarily on the individual biomarker performance, particularly GDF-15.

### 3.5. Biomarker Correlation

Exploratory correlation analyses were performed to evaluate associations between circulating mitochondrial stress biomarkers and selected biochemical parameters (Table 3). GDF-15 showed weak positive correlations with lactate (r = 0.339, *p* < 0.01) and ammonia (r = 0.437, *p* < 0.01). No significant associations were observed between GDF-15 and other biochemical parameters, including aspartate aminotransferase (AST), alanine aminotransferase (ALT), alkaline phosphatase (ALP) and glucose. In contrast, FGF-21 demonstrated weak correlations with glucose (r = 0.202, *p* = 0.042) and ALP (r = −0.255, *p* = 0.005), while no significant correlations were observed with lactate, AST, ALT or ammonia.

A moderate positive correlation was also observed between GDF-15 and FGF-21 concentrations across the entire cohort (r = 0.333, *p* < 0.001). When analysed by subgroup, the association remained significant in Group 1 (High-risk cohort; r = 0.309, *p* = 0.031) and Group 2 (Screen-positive/non-high-risk cohort; r = 0.463, *p* < 0.001), but was not observed in Groups 3–5. Overall, the correlations between mitochondrial stress biomarkers and routine biochemical parameters were modest, indicating that GDF-15 and FGF-21 may reflect mitochondrial stress responses that are not fully captured by conventional biochemical markers.

## 4. Discussion

This study evaluated the screening and stratification utility of GDF-15 and FGF-21 in individuals evaluated for suspected mitochondrial disease within a heterogeneous Malaysian referral cohort. By analysing circulating blood specimens (Groups 1, 2 and 4) together with exploratory fibroblast lysate specimens (Groups 3 and 5) across heterogeneous referral cohorts, our findings reflect biomarker performance in a real-world diagnostic setting rather than by genetically confirmed populations alone.

Both biomarkers were elevated in clinically high-risk and metabolically screen-positive individuals compared with healthy controls. However, GDF-15 demonstrated better discriminatory performance, reflected by a higher area under the ROC curve and more favourable sensitivity and specificity. Similar observations have been reported in recent comparative studies, in which GDF-15 showed stronger overall screening performance than FGF-21 across mixed mitochondrial disease cohorts [2,4]. Although GDF-15 demonstrated better overall performance than FGF-21, the observed AUC values remained within the moderate range, indicating that these biomarkers should not be interpreted as standalone diagnostic tools.

The relatively high sensitivity observed for GDF-15 at the selected screening threshold suggests potential utility as a referral triage biomarker rather than a disease-specific diagnostic marker. This distinction is important because elevation in both biomarkers has been described in secondary mitochondrial dysfunction and other systemic conditions, including infection, cardiac diseases and metabolic stress [6]. This reflects biological overlap across disease states and supports the role of these biomarkers as non-specific indicators of cellular stress.

The screen-positive/non-high-risk group represented a clinically heterogeneous population and included individuals with secondary lactic acidosis and abnormalities detected through metabolic screening investigations. These conditions are functionally related to mitochondrial energy metabolism and were included to reflect real-world referral practice. However, biomarker elevation within this group may reflect broader metabolic stress responses rather than primary mitochondrial disease alone. Subgroup-specific statistical analysis was not performed because the number of individuals within each metabolic category was insufficient for reliable interpretation.

Biomarker performance was also lower in the screen-positive/non-high-risk cohort, which likely represents a diagnostically ambiguous referral population encountered during routine metabolic evaluation. Clinically, this limits their ability to distinguish primary mitochondrial disease from other conditions. Therefore, these biomarkers should be used cautiously and only as adjuncts to clinical and laboratory evaluation in this subgroup. The lower AUC, sensitivity and specificity observed in this group suggest that GDF-15 and FGF-21 may have reduced discriminatory utility in individuals without classical neurological or multisystem features suggestive of mitochondrial disease.

FGF-21 demonstrated lower screening performance overall. Relatively elevated biomarker concentrations were observed in the single post-mortem fibroblast case with confirmed OXPHOS deficiency, although interpretation remains limited due to the extremely small number of confirmed cases. Previous studies have reported higher FGF-21 concentrations in disorders involving respiratory chain impairment, particularly in muscle-predominant phenotypes [16,17]. However, the present study was not designed to evaluate biomarker performance in genetically or biochemically confirmed mitochondrial disease alone.

A moderate positive correlation was observed between circulating GDF-15 and FGF-21 concentrations across the overall cohort, although the strength of association was modest. This suggests that both biomarkers may reflect overlapping but not identical mitochondrial stress signalling pathways. Previous studies have similarly reported variable correlations between these markers, indicating that they may respond to distinct aspects of mitochondrial dysfunction and cellular stress responses rather than functioning as interchangeable indicators [18,19]. Although both biomarkers reflect mitochondrial stress responses, combined interpretation did not improve discrimination compared with GDF-15 alone in the present cohort.

Correlation analysis with routine biochemical parameters demonstrated that GDF-15 showed weak positive associations with lactate and ammonia, whereas FGF-21 correlated modestly with glucose and alkaline phosphatase. These findings suggest that mitochondrial stress biomarkers capture metabolic disturbances that are only partially reflected by conventional clinical chemistry markers. The observed correlations were generally weak and should therefore be interpreted cautiously.

GDF-15 and FGF-21 are stress-responsive biomarkers associated with impaired mitochondrial energy metabolism. Increased circulating concentrations may reflect activation of systemic metabolic stress pathways during mitochondrial dysfunction [18,19]. However, both biomarkers may also be elevated in non-mitochondrial inflammatory or metabolic conditions, which limits disease specificity.

Importantly, GDF-15 and FGF-21 represent downstream stress-responsive biomarkers rather than direct measures of mitochondrial respiratory chain activity. Mitochondrial dysfunction may arise from diverse upstream abnormalities involving mitochondrial DNA maintenance, respiratory chain defects, or disturbances in mitochondrial fusion and fission dynamics, which subsequently activate integrated metabolic stress pathways [20,21]. Consequently, elevated biomarker concentrations should be interpreted as probabilistic indicators of mitochondrial stress rather than definitive evidence of primary OXPHOS dysfunction. The weak correlations observed in this study suggest that these biomarkers may reflect broader cellular stress responses that are not fully captured by routine biochemical parameters.

This study also included exploratory fibroblast lysate biomarker measurements alongside plasma analysis. Cellular biomarker assessment captures intracellular stress responses and may provide supplementary insight in post-mortem or tissue-based investigations. Although relatively few studies have incorporated fibroblast biomarker analysis into screening frameworks, emerging work suggests that integrating circulating and cellular data may provide interpretive context in diagnostically complex cases [5].

From a clinical implementation perspective, our findings are particularly relevant to resource-limited settings, including low- to medium-income countries in Southeast Asia, where access to genomic sequencing, respiratory chain enzyme analysis and specialised mitochondrial diagnostics remains constrained [11,12,13,14]. In such environments, minimally invasive biomarkers capable of supporting referral prioritisation may reduce diagnostic delay and optimise allocation of advanced testing resources. Nevertheless, biomarker interpretation must remain clinically contextual and integrated with standard biochemical and genetic investigations.

Studies from other Asian populations, including cohorts from China and Hong Kong, have similarly reported better overall screening performance for GDF-15 compared with FGF-21 in heterogeneous mitochondrial disease populations [5,12]. The present Malaysian cohort therefore appears broadly consistent with regional observations, although direct comparison remains limited by differences in study design, referral criteria and case confirmation strategies.

This study has several limitations. Most individuals in the cohort did not have molecular confirmation of mitochondrial disease. As a result, the ROC analysis reflects discrimination between clinically defined referral groups and controls rather than true diagnostic accuracy against a genetic gold standard and some degree of misclassification may be present. The use of archived referral specimens may also limit generalisability and introduce variability related to sample availability, handling and storage. In addition, the screen-positive/non-high-risk group was clinically heterogeneous and may include individuals with secondary mitochondrial dysfunction or non-specific metabolic stress. Fibroblast-based findings should be interpreted cautiously due to the small number of control cell lines. Potential confounding factors such as age, BMI, inflammatory status and intercurrent illness could not be fully evaluated due to incomplete data, and therefore non-mitochondrial contributions to GDF-15 elevation cannot be excluded.

In summary, GDF-15 and, to a lesser extent, FGF-21 may function as adjunct biomarkers that support early screening and stratification of individuals evaluated for suspected mitochondrial disease. Their primary utility appears to lie in referral triage and prioritisation for further metabolic or molecular evaluation rather than definitive diagnosis, particularly within heterogeneous clinical populations.

## Figures and Tables

**Table 1:** Demographic characteristics and GDF-15 and FGF-21 concentrations across study groups.

Variable		Group					
		Total	High Risk	Non-High Risk	Post-Mortem (Fibroblast)	Healthy Controls (Plasma)	Healthy Controls (Fibroblast)
		*n* = 221	*n* = 49	*n* = 111	*n* = 13	*n* = 45	*n* = 3
Age	Early childhood (<2 years)	148	47	88	13	0	0
	Children (3–12 years)	23	1	21	0	0	1
	Juvenile (13–18 years)	2	0	2	0	0	0
	Adults (>19 years)	48	1	0	0	45	2
Gender	Male	123	30	66	9	16	2
	Female	98	19	45	4	29	1
Ethnicity	Malay	176	34	92	10	38	2
	Chinese	19	6	9	3	1	0
	Indian	10	3	5	0	2	0
	Aborigines/Native	15	6	5	0	4	0
	Other	1	0	0	0	0	1
Clinical presentation	Organomegaly	20	6	14	0	0	0
	Failure to thrive	24	3	21	0	0	0
	Epilepsy	60	16	43	1	0	0
	Muscle weakness	31	12	19	0	0	0
	Developmental delay	34	5	29	0	0	0
	Cardiomyopathy	5	2	3	0	0	0
	Lethargy	9	2	7	0	0	0
	Hypoglycaemia	10	2	8	0	0	0
	Septicaemia	21	9	12	0	0	0
	Intellectual disability	12	9	3	0	0	0
	Hypotonia	21	9	12	0	0	0
GDF-15 (pg/mL)	Mean (standard deviation)	-	-	-	-	-	39.39 ± 44.79
	Median (inter quartile range)	271.42 (1147.75)	4666.85 (42,319.3)	318.67 (720.5)	12.65 (57.56)	235.43 (98.79)	-
FGF-21 (pg/mL)	Mean (standard deviation)	-	-	-	-	-	34.39 ± 18.43
	Median (inter quartile range)	66.96 (262.09)	202.69 (2879.82)	73.89 (273.25)	221.21 (312.94)	38.98 (48.04)	-

Note: Categorical variables are presented as counts (*n*). Continuous variables are presented as median (interquartile range, IQR) or mean ± standard deviation (SD) according to data distribution. Descriptive statistics were used to summarise demographic characteristics and biomarker concentrations across study groups.

**Table 2:** Screening performance of GDF-15 and FGF-21 at cut-off values of 300 pg/mL and 55 pg/mL, respectively.

Parameter	GDF-15		FGF-21	
	Group 1	Group 2	Group 1	Group 2
Sensitivity	90.24%	59.05%	82.93%	58.85%
Specificity	75.56%	75.56%	68.89%	68.79%
Positive Likelihood Ratio	3.69	2.42	2.67	1.89
Negative Likelihood Ratio	0.13	0.54	0.25	0.6
Disease Prevalence	47.67%	70.00%	46.67%	69.80%
Positive Predictive Value	77.08%	84.93%	70.83%	81.33%
Negative Predictive Value	89.47%	44.16%	81.58%	41.89%
Area Under Curve	0.7187	0.6008	0.6301	0.5465
Standard Error	0.0556	0.0489	0.0603	0.0508

**Table 3:** Spearman correlation between mitochondrial stress biomarkers and biochemical parameters.

Biochemical Parameters	GDF-15		FGF-21	
	r	*p*-Value	r	*p*-Value
Lactate	**0.339**	**<0.01**	0.043	0.614
Ammonia	**0.437**	**<0.01**	0.110	0.265
Glucose	0.151	0.133	**0.202**	**0.042**
Aspartate aminotransferase (AST)	0.215	0.070	0.084	0.482
Alanine aminotransferase (ALT)	0.043	0.643	0.071	0.443
Alkaline phosphatase (ALP)	−0.138	0.134	**−0.255**	**0.005**

Note: Correlations were assessed using Spearman’s rank correlation analysis. Significant correlations (*p* < 0.05) are shown in bold.

## Data Availability

The datasets generated and analysed during the current study are available in the NIH Data Repository System (NIH-DaRS), Ministry of Health Malaysia (https://nihdars.nih.gov.my, accessed on 24 February 2025). Data are available under restricted access and may be obtained upon reasonable request and approval through the NIH-DaRS data access procedure.

## Abbreviations

The following abbreviations are used in this manuscript:

AUC	Area under the curve
ELISA	Enzyme-linked immunosorbent assay
FGF-21	Fibroblast growth factor 21
GDF-15	Growth differentiation factor 15
IEM	Inborn errors of metabolism
MS/MS	Tandem mass spectrometry
OXPHOS	Oxidative phosphorylation
ROC	Receiver operating characteristic
SPSS	Statistical Packages for the Social Sciences
